# Exploring the influence of gender, seniority and specialty on paper and computer-based feedback provision during mini-CEX assessments in a busy emergency department

**DOI:** 10.1007/s10459-016-9682-9

**Published:** 2016-04-25

**Authors:** Yu-Che Chang, Ching-Hsing Lee, Chien-Kuang Chen, Chien-Hung Liao, Chip-Jin Ng, Jih-Chang Chen, Chung-Hsien Chaou

**Affiliations:** 1 Chang Gung Medical Education Research Center, CGMERC, No. 5, Fusing St., Gueishan Township, 333 Taoyuan city, Taiwan (R.O.C.); 2 Department of Emergency Medicine, Chang Gung Memorial Hospital, Linkou, and Chang Gung University College of Medicine, Taoyuan City, Taiwan (R.O.C.); 3 Department of Medical Education, Chang Gung Memorial Hospital, Linkou, Taoyuan city, Taiwan (R.O.C.); 4 Department of Traumatology and Emergency Surgery, Chang Gung Memorial Hospital, Linkou, and Chang Gung University College of Medicine, Taoyuan city, Taiwan (R.O.C.)

**Keywords:** mini-CEX, Feedback, Emergency department, Residency training, Computer-based format

## Abstract

The mini-clinical evaluation exercise (mini-CEX) is a well-established method of assessing trainees’ clinical competence in the workplace. In order to improve the quality of clinical learning, factors that influence the provision of feedback are worthy of further investigation. A retrospective data analysis of documented feedback provided by assessors using the mini-CEX in a busy emergency department (ED) was conducted. The assessors comprised emergency physicians (EPs) and trauma surgeons. The trainees were all postgraduate year one (PGY1) residents. The completion rate and word count for each of three feedback components (positive feedback, suggestions for development, and an agreed action plan) were recorded. Other variables included observation time, feedback time, the format used (paper versus computer-based), the seniority of the assessor, the gender of the assessor and the specialty of the assessor. The components of feedback provided by the assessors and the influence of these contextual and demographic factors were also analyzed. During a 26-month study period, 1101 mini-CEX assessments (from 273 PGY1 residents and 67 assessors) were collected. The overall completion rate for the feedback components was 85.3 % (positive feedback), 54.8 % (suggestions for development), and 29.5 % (agreed action plan). In only 22.9 % of the total mini-CEX assessments were all three aspects of feedback completed, and 7.4 % contained no feedback. In the univariate analysis, the mini-CEX format, the seniority of the assessor and the specialty of the assessor were identified as influencing the completion of all three components of feedback. In the multivariate analysis, only the mini-CEX format and the seniority of the assessor were statistically significant. In a subgroup analysis, the feedback-facilitating effect of the computer-based format was uneven across junior and senior EPs. In addition, feedback provision showed a primacy effect: assessors tended to provide only the first or second feedback components in a busy ED setting. In summary, the authors explored the influence of gender, seniority and specialty on paper and computer-based feedback provision during mini-CEX assessments for PGY1 residency training in a busy ED. It was shown that junior assessors were more likely to provide all three aspects of written feedback in the mini-CEX than were senior assessors. The computer-based format facilitated the completion of feedback among EPs.

## Introduction

The mini-clinical evaluation exercise (mini-CEX) is a well-known reliable method of assessing trainees’ clinical competence in the workplace (Kogan et al. 2009; Al Ansari et al. 2013; Alves de Lima et al. 2013). It was introduced into the Taiwanese medical education system 10 years ago and has been widely integrated into medical education curriculums since then (Chen et al. 2005; Liao et al. 2013). Uniquely, it is the number and breadth of the structured feedback comments that make the mini-CEX a rich assessment tool (Pernar et al. 2011). Receiving appropriate feedback promotes the identification of strengths and weaknesses within trainees’ clinical competencies, which is crucial for effective teaching and learning (Carr 2006). Previous studies have documented the advantages of feasibility and utility of the mini-CEX for promoting feedback in a clinical workplace setting (Kogan et al. 2002; Wiles et al. 2007; Weller et al. 2009). However, not all trainees report receiving useful feedback, and in some cases, feedback provided by assessors may be poor (Fernando et al. 2008; Cohen et al. 2009). Thus, in order to improve the quality of clinical learning, factors that influence the provision of feedback are worth further investigation.

The digitalization of assessment tools in order to make workplace teaching and learning more efficient has become a worldwide trend. One randomized controlled study found that by modifying the format and process of the assessment tool, more recorded observations were made and assessor accuracy improved (Donato et al. 2008). Furthermore, both personal digital assistant (PDA)- and paper-based mini-CEX assessments have demonstrated high reliability (Torre et al. 2011). However, studies examining the correlation between digitalization and the provision of feedback in mini-CEX assessments are few (Torre et al. 2007, 2011). In this study, we aimed to explore the factors influencing the provision of feedback, including the influence of a computer-based format on the provision of feedback.

## Materials and methods

### Study design

Our study comprised a retrospective data analysis of the influence of contextual and demographic factors on the provision of feedback by assessors using the mini-CEX to evaluate the clinical competency of trainees in an emergency department (ED). The evaluations were conducted in the ED of Chang Gung Memorial Hospital (CGMH), a 3800-bed tertiary hospital in Taoyuan, Taiwan. This study was approved by the Institutional Review Board of Chang Gung Memorial Hospital.

### Participants

The assessors comprised emergency physicians (EPs) and trauma surgeons. The trainees were all postgraduate year one (PGY1) residents. According to the regulations of the Ministry of Health, all medical students should have a 1-year rotation after graduation and before choosing their own specialty. In addition to emergency medicine, the one-year rotation curriculum also includes internal medicine, gynecology/obstetrics, pediatrics, general surgery, community medicine and a number of months in the specialty of their choosing. Each PGY1 resident received a curriculum of 1 week of trauma emergency training and 3 weeks of non-trauma emergency training during the one-month ED rotation. A total of 67 assessors (50 EPs and 17 trauma surgeons) evaluated 273 PGY1 residents during their emergency medicine training.

### Data collection

The data was collected between November 2009 and December 2011. The mini-CEX used was in a traditional Chinese format that has been previously validated (Chen et al. 2006). In the mini-CEX, a single assessor observes the trainee conducting a focused history-taking and physical examination. The trainee then presents any differential diagnoses and/or a management plan. The seven itemized clinical competencies (medical interviewing, physical examination, clinical skills, counseling skills, clinical judgment, organization/efficiency, and professionalism) are rated using a nine-point rating scale (1 = unsatisfactory and 9 = superior). This scaling was used as it is believed to provide greater accuracy (Cook and Beckman 2009). In our study, feedback was given to the trainees at the end of the mini-CEX as part of the workplace assessment. Feedback included three components: positive feedback (particular strengths), suggestions for development, and an agreed educational plan of action. Feedback time was estimated by the assessor and recorded on the mini-CEX form. The mini-CEX was completed weekly to evaluate the clinical competency of PGY1 doctors. However, PGY1 doctors were allowed to request additional mini-CEXs if they wished to receive more assessment and feedback on their performance during the ED rotation.

For the first 9 months, the mini-CEX and the feedback were provided to all trainees using a paper-based format. Over the following 17 months, the mini-CEX and feedback provided by the EPs shifted to a computer-based format, while those provided by the trauma surgeons remained in the paper-based format. The computer-based format was not provided for trauma surgeons due to lack of funding. The content of the different formats was identical.

When a computer-based format was used, immediately after evaluating the patient, the assessor and the trainee sat in front of a computer that ran the ED teaching support software. The computer format was easily accessible for EPs in the emergency department. The mini-CEX data were collected by a program assistant in the residency training office using a standard procedure.

### Data analysis

We analysed the mean age of patients, the seniority of the assessor, the observation time, the feedback time, the gender of the assessor, the specialty of the assessor, the completion rate of each domain of clinical competency, the word count (in Chinese) used for feedback on each component, and the impact of the format used on the provision of all three aspects of feedback. We also analyzed the frequency of use for each feedback component (positive feedback, suggestions for development and an agreed plan of action), of all feedback components and of no feedback for weekly mini-CEXs. When comparing results between two groups, where appropriate, a *χ* ^2^ test or a Fisher’s exact test was applied for the categorical data. A two-tailed independent *t* test was used for the continuous variables. Bonferroni correction was applied to adjust multiple comparisons. Univariate and multivariate logistic regressions were used to identify possible correlates influencing the provision of all three aspects of feedback. Data were analyzed using SAS statistical software, version 9.2 (SAS Institute Inc., Cary, NC). Alpha was set at *p* < 0.05.

## Results

### Overall

A total of 1101 mini-CEX ratings of PGY1 residents were completed during the study period. During the study period, EPs rated 899 PGY1-patient encounters and trauma surgeons rated 202 PGY1-patient encounters. The EP-rated mini-CEXs comprised 295 paper-format evaluations and 604 computer-format evaluations. The overall completion rates for the components of positive feedback, suggestions for development and agreed action plan were 85.3, 54.8, and 29.5 %, respectively. Only 22.9 % of the total mini-CEXs contained all three components of feedback, while 7.4 % of mini-CEXs were without any feedback.

### Quality analysis of feedback completed by faculties with different specialties

Feedback was provided on 878 (97.7 %) mini-CEXs rated by emergency physicians and on 142 (70.3 %) mini-CEXs rated by trauma surgeons. Using the paper format of the mini-CEX in the ED, the feedback time was longer when PGY1 residents were rated by trauma surgeons than when they were rated by EPs (Table 1). Except for clinical skills, the completion rate for other domains of clinical competency was similar for the EPs and trauma surgeons when the paper-based format was used.

**Table 1:** Demographics and comparison of mini-CEX components by different assessor specialties and different formats

	Overall (n = 1101)	Emergency physician			Trauma surgeon	
		Paper format (n = 295)	Computer format (n = 604)	*p* value	Paper format (n = 202)	*p* value^c^
Mean age of patients^b^	52.2 (20.8)	55.1 (20.3)	54.3 (19.3)	0.583	41.7 (23.2)	<.001*
Assessor seniority (years)^b^	7.32 (5.05)	9.46 (4.88)	7.80 (4.58)	<.001*	2.46 (3.37)	<.001*
Observation time (min)^b^	14.9 (10.9)	14.0 (6.36)	14.8 (8.61)	0.140	17.9 (23.4)	0.092
Feedback time (min)^b^	11.1 (5.76)	10.5 (6.72)	11.1 (5.03)	0.169	12.5 (6.99)	0.010*
Male sex^a^						
Examinee	731 (66.4)	189 (64.1)	411 (68.1)	0.235	131 (64.9)	0.858
Assessor	984 (89.4)	268 (90.1)	514 (85.1)	0.016*	202 (100)	<.001*
Clinical domains measured^a^						
Medical interview	1096 (99.6)	294 (99.7)	603 (99.8)	0.549	199 (98.5)	0.309
Physical examination	1088 (98.8)	291 (98.6)	600 (99.4)	0.450	197 (97.5)	0.496
Clinical skills	367 (33.3)	144 (48.8)	72 (11.9)	NA^d^	151 (74.8)	NA^d^
Counselling skills	979 (88.9)	265 (89.8)	533 (88.3)	0.480	181 (89.6)	0.935
Clinical judgment	1091 (99.1)	292 (98.9)	600 (99.3)	0.690	199 (98.5)	0.691
Efficiency/Organized	1067 (96.9)	279 (94.6)	599 (99.2)	<.001*	189 (93.6)	0.636
Professionalism	1060 (96.3)	277 (93.9)	600 (99.3)	<.001*	183 (90.6)	0.168
Word counts for each component^b^						
Positive feedback	9.80 (6.94)	11.4 (7.31)	8.98 (6.62)	<.001*	10.0 (6.92)	0.071
Suggestions for development	10.6 (6.99)	11.0 (6.21)	10.6 (7.55)	0.504	9.24 (5.33)	0.040
Agreed action plan	11.6 (7.95)	9.20 (5.10)	12.8 (8.88)	<.001*	8.88 (4.15)	0.771
Feedback components used^a^						
Positive feedback	939 (85.3)	263 (89.2)	540 (89.4)	0.910	136 (67.3)	<.001*
Suggestions for development	603 (54.8)	168 (57.0)	368 (60.9)	0.254	67 (33.2)	<.001*
Agreed action plan	325 (29.5)	83 (28.1)	216 (35.8)	0.023*	26 (12.9)	<.001*
All aspects of feedback provided^a^	252 (22.9)	56 (19.0)	171 (28.3)	0.003*	25 (12.4)	0.050
No aspects of feedback provided^a^	81(7.36)	15 (5.08)	6 (0.99)	<.001*	60 (29.7)	<.001*

*NA* non-accessible

* Statistically significant (*p* < 0.025). Significance level adjusted by Bonferroni method

^a^Data presented as number (%)

^b^Data presented as mean (SD)

^c^Compare paper format used by EPs or Trauma surgeons

^d^DOPS (Direct observation of procedural skill) was implemented to assist assessment of ED learner’s competence on procedure skills, which gave rise to the reduction of frequency of assessing technical skills

The word counts of the feedback for each of the three components were similar for EPs and trauma surgeons. However, the absolute percentages of the three feedback components used (positive feedback, suggestions for development and an agreed action plan) were higher for the EP group: 89.2, 57.0 and 28.1 %, respectively when provided by EPs; and 67.3, 33.2 and 12.9 %, respectively when provided by trauma surgeons. Feedback containing all three components was provided on 19.0 % of the assessments by EPs and on 12.4 % of those completed by trauma physicians. Only 5.1 % of assessments completed by EPs, but 29.7 % of those by trauma surgeons, provided no feedback at all.

### Comparing feedback on mini-CEXs using different formats

After a computer-based mini-CEX was implemented, EPs were requested to complete mini-CEXs in this format. The computer-based format contained exactly the same components as the paper-based format. The mean age of patients, observation time, feedback time and gender of the examinees were similar for both formats. However, the seniority of the assessor was greater and a higher proportion of men were in the group using the paper-based format. As it was decided that PGY1 doctors would be free to choose their assessors in the clinical placement setting, a selection bias could have resulted due to their choices.

In the clinical domain of competency, efficiency/organization and professionalism were evaluated more often using the computer-based format than using paper-based format (94.6 vs. 99.2 %, *p* < 0.001 and 93.9 vs. 99.3 %, *p* < 0.001, respectively). Clinical skills were evaluated more often using the paper-based format than using computer-based format (48.8 vs. 11.9 %). The difference between clinical skills’ results and other results is related to the interference of the implementation of direct observation of procedural skill (DOPS), which explained the reduction in the frequency of assessing technical skills in the mini-CEX. Therefore, it made no sense to compare the completion rates for the clinical skill assessment across the paper- and computer-based different formats.

EPs who used the paper-format mini-CEX provided more feedback for positive feedback than those used the computer-based format (a mean word count of 11.40 ± 7.31 vs. 8.98 ± 6.62, *p* < 0.001), while more feedback was provided using the computer-based format than paper-based format for agreed action plans (mean word count 12.8 ± 8.88 vs. 9.2 ± 5.1, *p* < 0.001; frequency of feedback component used 35.8 % vs. 28.1 %, *p* = 0.023). Feedback containing all three components was provided by 28.3 % of the mini-CEXs using the computer-based format and 19.0 % of mini-CEXs using the paper-based format (Table 1).

### Factors associated with the provision of all aspects of feedback

The factors associated with the provision of all aspects of feedback during a mini-CEX evaluation in an ED are shown in Table 2. In the univariate analysis, the seniority of the assessor [odds ratio (OR) 0.41; 95 % CI 0.29–0.58], the mini-CEX format (OR 2.03; 95 % CI 1.51–2.73) and the specialty of the assessor (OR 2.39; 95 % CI 1.53–3.73) were statistically significant. However, in the multivariate logistic regression in which all covariates were adjusted, only the seniority of the assessor (OR 0.35; 95 % CI 0.24–0.51) and the mini-CEX format (OR 1.47; 95 % CI 1.01–2.15) were statistically significant. The results suggest that a computer-format evaluation and physicians with fewer than 10 years of seniority are more likely to complete all three aspects of feedback.

**Table 2:** Variates analysis of factors affecting the provision of all three aspects of feedback

	Univariate analysis		Multivariate analysis	
	Coefficient	OR (95 % CI)	Coefficient	OR (95 % CI)
Gender of assessor				
Male	Reference		Reference	
Female	0.411	1.509 (0.988–2.305)	−0.105	0.900 (0.547–1.411)
Seniority				
Fewer than 10 years	Reference		Reference	
More than 10 years	−0.892*	0.410 (0.289–0.584)	−1.053*	0.349 (0.238–0.510)
Mini CEX format (nested in EP level)				
Paper	Reference		Reference	
Computer	0.707*	2.028 (1.508–2.729)	0.387*	1.473 (1.007–2.153)
Specialty of assessor				
Trauma surgeon	Reference		Reference	
Emergency physician	0.872*	2.392 (1.533–3.732)	0.189	1.208 (0.667–2.186)
Observation time	0.003	1.003 (0.991–1.016)	0.004	1.004 (0.991–1.016)

* Statistically significant (*p* < 0.05)

### Subgroup analysis of age versus computer format

We further conducted a subgroup analysis of emergency physicians who used computer formats to determine the difference between junior and senior EPs in using computers as rating tools. We categorized EPs into junior and senior groups by seniority with a cut-off point of 10 years. Longer feedback times were observed for mini-CEXs provided by junior EPs than for those by seniors. Junior EPs also provided a significantly higher portion of all three aspects of feedback (37.1 %) than did seniors (8.60 %, *p* < 0.001) when using the computer format. Two of the feedback components, suggestions for development and an agreed action plan, were also used significantly more often by junior EPs than by seniors (Table 3).

**Table 3:** The subgroup analysis of feedback provided by emergency physician using computer-format mini-CEX

	Junior EPs	Senior EPs	*p* value
	N = 418	N = 186	
Mini-CEX time (in minutes)^a^			
Observation time	14.9 ± 9.6	14.4 ± 5.7	0.347
Feedback time	11.6 ± 5.2	9.93 ± 4.4	<0.001
The frequency of each component utilized for feedback^b^			
Positive feedback	375 (89.7)	165 (88.7)	0.712
Suggestions for development	289 (69.1)	79 (42.5)	<0.001
Agreed action plan	170 (40.7)	46 (24.7)	<0.001
All three aspects of feedback provided^b^	155 (37.1)	16 (8.60)	<0.001

^a^Data presented as mean ± SD. Comparison between the two groups using independent *t* test

^b^Data presented as number (%). Comparison between the two groups Chi square test

## Discussion

Recently, it has been continually emphasized in the literature that formative assessments such as the mini-CEX could be used as a tool for optimizing learning in a medical education context (Schuwirth 2013). The health professions are expected to nurture recipient reflection-in-action for achieving truly effective feedback (Archer 2010). However, fewer than a quarter of the mini-CEXs (22.9 %) recorded all aspects of feedback in our study. Moreover, some of the mini-CEXs were void of feedback, probably due to our busy ED setting. Indeed, Kogan et al. (2012) pointed out that provision of feedback is a complex and dynamic process influenced by many factors. Previous studies have also shown that provision of effective feedback in healthcare education can be problematic and that some barriers to giving feedback exist, namely, under using interactive the feedback methods of self-assessment and action plans (Holmboe et al. 2004; Colthart et al. 2008). Some new models of feedback in higher and professional education have addressed the impact of culture and continuum on complex and contextual feedback (Archer 2010; Boud and Molloy 2013) Therefore, we decided to explore the influence of contextual and demographic factors on the provision of feedback during mini-CEX assessments in an emergency department.

Several factors have been identified as influential in giving feedback, such as the specialty, the assessor group, the self-confidence of the assessor in his or her clinical and feedback skills and whether or not the assessor is an academic trainee (Fernando et al. 2008; Kogan et al. 2012). In our study, the seniority of the assessor and the computer-based format used were considered individually as negative and positive influential factors in giving feedback to learners in the ED. Compared with the itemized rating section, providing written feedback is usually more time-consuming. Li et al. (2016) evaluated EP efficiency in the Taiwanese healthcare system. They found that senior EPs take longer than junior EPs when ordering prescriptions and patient disposition in treating both urgent and non-urgent patients (Li et al. 2016). It is likely that some of the written feedback components were possibly omitted for these reasons in our busy ED.

No previous studies have been conducted to directly compare feedback obtained via different formats. Our results suggest that a computer-based format facilitates the provision of feedback in a mini-CEX evaluation across EPs. Indeed, focusing on the rating process rather than on the feedback, Torre et al. (2011) found similar results following the digitalization of the mini-CEX format. However, the rating process requires considerably less typing/writing than does the feedback process. Since for most people, typing is faster than handwriting, our results favor the use of a computer-based format for faster feedback provision. This would have obvious benefits in a busy ED setting for EPs and might also be applicable for mini-CEX assessments in other disciplines.

In our study, the electronic format of the mini-CEX had an uneven effect on facilitating feedback from senior and junior assessors. Feedback time and most of the completion rates were significantly higher among junior EPs than among seniors. Similar results have also been found in previous studies comparing mini-CEXs with paper-based and personal digital assistant (PDA)-based formats (Torre et al. 2007, 2011). This is possibly because junior faculty are likely to be more familiar with the operation of a computer interface in the assessment and feedback process than are seniors. From this perspective, we propose that, although the computer format generally facilitates the provision of the feedback, some barriers to its usage among senior assessors may exist (McLeod et al. 2003; Torre et al. 2011).

**Fig. 1:**
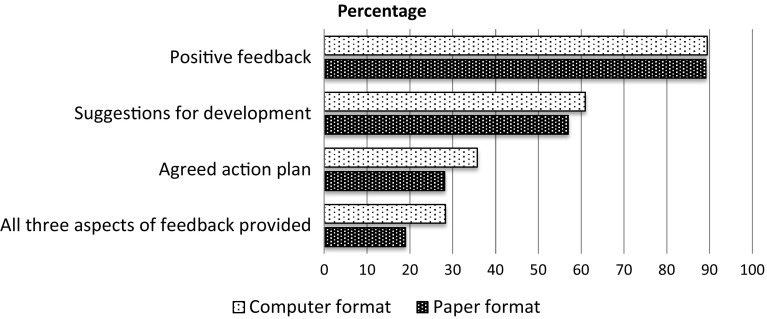
A primacy effect is shown in the completion rate of feedback components in mini-CEX assessments. The completion rate of each feedback component decreased in accordance with its order of presentation in the mini-CEX evaluation forms

In this study, approximately 85.3 % of the assessors completed the complimentary part of the feedback, positive feedback. In contrast, only 54.8 % provided suggestions for development and 29.5 % provided an agreed action plan individually. The completion rate of each feedback component decreased in accordance with its order of presentation, which has also been found in previous studies (Fernando et al. 2008; Pelgrim et al. 2012). This phenomenon in our study could be a primacy effect: in a busy ED setting, some assessors tend to complete only the first or second feedback component, especially in situations of clinical overload or high stress. This can be inferred from Fig. 1. A previous study also revealed that the observation and evaluation times allotted to an EM faculty member are often limited (Chisholm et al. 2004). Another possible explanation is that, culturally, most people are unwilling to point out another’s weaknesses face to face, even if such an interaction takes place between a teacher and a trainee (Colletti 2000).

## Limitations

This study has several limitations. First, it is a retrospective data analysis and a selection bias could have resulted. Second, we did not incorporate environmental factors, such as ED crowding and the emergency physician’s clinical load. These factors are difficult to quantify but can influence the quality of a mini-CEX assessment. Third, we simply quantified the completion rate and word count of the feedback given by the assessors, instead of qualitatively analyzing the contents of the feedback. One could argue that the provision of all three components might not necessarily equate to high-quality feedback. Fourth, the trauma surgeons did not start with the computer format due to lack of funding. Fifth, it was decided that PGY1 trainees would be free to choose their assessors in the clinical placement setting; accordingly, a selection bias could have resulted. Sixth, the implementation of direct observation of procedural skill (DOPS) explains a reduction in the frequency of assessing technical skills in the mini-CEX. Therefore, it made no sense to compare the completion rate of clinical skill assessment using different formats. Finally, it is very likely that some of the communication between trainees and assessors was not documented in the mini-CEX form; further investigation is needed to evaluate the gap between verbal feedback and written feedback.

## Conclusion

Our study analyzed the provision of feedback in mini-CEX assessments during PGY1 resident ED rotations. Junior assessors were more likely to provide all three aspects of feedback than were senior assessors. A computer-based format facilitated the completion of feedback, especially on the part of junior assessors among EPs. Additional studies are needed to qualitatively analyze differences and to consider the effects of computer-based formats used by other specialized doctors.
